# Cross-Sectional Associations between Multiple Lifestyle Behaviors and Health-Related Quality of Life in the 10,000 Steps Cohort

**DOI:** 10.1371/journal.pone.0094184

**Published:** 2014-04-08

**Authors:** Mitch J. Duncan, Christopher E. Kline, Corneel Vandelanotte, Charli Sargent, Naomi L. Rogers, Lee Di Milia

**Affiliations:** 1 Centre for Physical Activity Studies, Institute for Health and Social Science Research, Central Queensland University, Rockhampton, Queensland, Australia; 2 Department of Psychiatry, University of Pittsburgh School of Medicine, Pittsburgh, Pennsylvania, United States of America; 3 Appleton Institute for Behavioural Science, Central Queensland University, Adelaide, South Australia, Australia; 4 School of Business and Law, Central Queensland University, Rockhampton, Queensland, Australia; 5 School of Business and Law and Institute for Health and Social Science Research, Central Queensland University, Rockhampton, Queensland, Australia; Hunter College, City University of New York (CUNY), CUNY School of Public Health, United States of America

## Abstract

**Background:**

The independent and combined influence of smoking, alcohol consumption, physical activity, diet, sitting time, and sleep duration and quality on health status is not routinely examined. This study investigates the relationships between these lifestyle behaviors, independently and in combination, and health-related quality of life (HRQOL).

**Methods:**

Adult members of the 10,000 Steps project (n = 159,699) were invited to participate in an online survey in November-December 2011. Participant socio-demographics, lifestyle behaviors, and HRQOL (poor self-rated health; frequent unhealthy days) were assessed by self-report. The combined influence of poor lifestyle behaviors were examined, independently and also as part of two lifestyle behavior indices, one excluding sleep quality (Index 1) and one including sleep quality (Index 2). Adjusted Cox proportional hazard models were used to examine relationships between lifestyle behaviors and HRQOL.

**Results:**

A total of 10,478 participants provided complete data for the current study. For Index 1, the Prevalence Ratio (p value) of poor self-rated health was 1.54 (p = 0.001), 2.07 (p≤0.001), 3.00 (p≤0.001), 3.61 (p≤0.001) and 3.89 (p≤0.001) for people reporting two, three, four, five and six poor lifestyle behaviors, compared to people with 0–1 poor lifestyle behaviors. For Index 2, the Prevalence Ratio (p value) of poor self-rated health was 2.26 (p = 0.007), 3.29 (p≤0.001), 4.68 (p≤0.001), 6.48 (p≤0.001), 7.91 (p≤0.001) and 8.55 (p≤0.001) for people reporting two, three, four, five, six and seven poor lifestyle behaviors, compared to people with 0–1 poor lifestyle behaviors. Associations between the combined lifestyle behavior index and frequent unhealthy days were statistically significant and similar to those observed for poor self-rated health.

**Conclusions:**

Engaging in a greater number of poor lifestyle behaviors was associated with a higher prevalence of poor HRQOL. This association was exacerbated when sleep quality was included in the index.

## Introduction

Engaging in one or more poor lifestyle behaviors such as smoking, risky alcohol consumption, physical inactivity and poor diet increases mortality risk [1]–[4]. These behaviors are associated with mortality risk in a dose-response manner as the number of poor behaviors increases [1]–[4]. Evidence also indicates an association between the time spent sitting and sleeping and poorer health status [3]–[9]. A greater number of hours spent sitting per day is associated with increased risk of chronic disease, CVD and all-cause mortality [7], [10], [11]. In addition, sleeping <7 or >8 hours per night is linked to increased risk of CVD and all-cause mortality [4], [12]. Studies have demonstrated that longer sitting duration *or* not sleeping 7 to 8 hours per night in combination with smoking, risky alcohol consumption, physical inactivity and poor diet, as part of a lifestyle behavior index, is associated with increased mortality risk in a dose-response manner [3], [4], [13], [14]. However, few studies have examined the association between health status and the aforementioned lifestyle behaviors when both sitting duration *and* not sleeping 7 to 8 hours per night are included in the analyses [4], [15]. This is important as many adults engage in *all* these lifestyle behaviors in ways that are considered to pose a risk to their health [1], [7], [10], [15]–[17].

Additionally, sleep duration is only one component of sleep behavior; sleep quality may also impact health status independent of sleep duration [6], [18]. Sleep quality can be defined as difficulty in falling asleep and/or remaining asleep and is commonly assessed in population studies by self-report and user ratings [19], [20]. Up to 41% of adults report sleep difficulties or poor sleep quality [6], [21], yet the impact of sleep quality in combination with a wide variety of other lifestyle behaviors is infrequently examined as a potential influence of health status. Self-rated health and health-related quality of life are overall indicators of health status that are predictive of mortality and chronic disease risk [22]. As such, they are useful indicators to understand how lifestyle behaviors influence the health status. Therefore, the purpose of this study was to 1) examine how smoking, risky alcohol consumption, physical inactivity, poor dietary behaviors, prolonged sitting time, not sleeping 7 to 8 hours per night and poor sleep quality are individually associated with poor self-rated health and frequent unhealthy days, and 2) examine the combined association of these behaviors with poor self-rated health and frequent unhealthy days in two indices, one excluding sleep quality and one including sleep quality.

## Methods

### Design and Participants

For this cross-sectional study, participants were members of the 10,000 Steps project. 10,000 Steps is a web-based (www.10000steps.org.au) physical activity promotion initiative where participants record the number of steps taken each day. A pedometer is used by participants to quantify their walking activity. The project was launched in 2001 and, as of November 2011 when this study was conducted, had 159,698 registered participants. Registered participants remain in the database but on average participants use the website for approximately 44 days [23]. In November 2011, all members were emailed an invitation to participate in an online survey of lifestyle behaviors, health outcomes and satisfaction with the 10,000 Steps project. This manuscript reports only those measures and results relevant to its purpose. The Central Queensland University Human Research Ethics Committee provided approval for the study and all participants provided informed consent to participate in the study via agreeing to take part in the online survey.

### Lifestyle Behavior Measures

A single item was used to classify participants as either a *current smoker* (at least one cigarette per day for the last month) or *non-smoker*. Alcohol consumption was assessed using two items: the frequency of consuming alcohol and the number of alcoholic drinks consumed on a day when alcohol was consumed. These items were used to classify participants into *lower risk* (≤2 drinks per day on a day when alcohol was consumed) or *higher risk* drinking (≥3 drinks per day on a day when alcohol was consumed). These criteria are similar to Australian guidelines for lower risk drinking over the lifetime [24]. Physical activity during the previous seven days was measured using the International Physical Activity Questionnaire Long Form (IPAQ-LF), a valid and reliable measure of walking, moderate and vigorous intensity physical activity undertaken in domestic, occupational, leisure and transport domains [25]. Standard IPAQ-LF scoring guidelines were applied and participants were classified into either *Low, Moderate* or *High* amounts of physical activity. Sitting time over the previous seven days on work and non-work days during travel activities, at work, watching TV, using a computer at home and during other leisure activities was measured using the Workforce Sitting Questionnaire, a valid and reliable instrument of sitting time [26]. Daily sitting time was calculated as the sum of sitting time reported during each activity on a work and non-work day based on the number of days worked in the last seven days [26]. Daily sitting time was subsequently classified into 3 categories (*<8 hours per day, ≥8 to <11 hours per day, ≥11 hours per day*), as these thresholds of sitting have previously been employed to demonstrate associations between sitting time and risk of all-cause mortality [7]. Sleep behaviors were measured using two items from the reliable and valid Pittsburgh Sleep Quality Index (PSQI) [27]: the duration of sleep each night over the past month (hours per night) and the overall sleep quality in the last month (very good, fairly good, fairly bad, very bad). Sleep duration was classified into three categories (*<7 hours per night, ≥7 to <8 hours per night, ≥8 hours per night*) based on the associations of not sleeping 7 to 8 hours per night with obesity, cardiovascular disease and all-cause mortality risk [4], [12], [28]. Sleep quality was categorized into three groups, *fairly bad* (very bad and fairly bad), *fairly good* and *very good* based on the distribution of the data and previous use of a trichotomous classification of sleep quality which demonstrated associations with quality of life [29]. Based on a previously used scale [30], dietary behaviors were assessed using four items that evaluated the daily frequency of fruit and vegetable consumption and the number of times soft drinks and fast foods were consumed in the previous 7 days [30]. Higher consumption of fruits and vegetables is associated with lower risk of obesity while greater soft drink and fast food consumption is associated with higher risk of obesity [30]; thus, the four measures were combined into a scale of overall dietary behaviors. Due to different recall periods of the four items, responses were converted to z-scores and summed together. The inverse of the z-scores for soft drink and fast food were used in the summary score of dietary behaviors so that a higher score reflected a better dietary quality. The dietary behaviors score was subsequently classified into tertiles (*Highest Tertile* (better diet), *Middle Tertile* (average diet), *Lowest Tertile* (poorer diet)). Participant socio-demographics assessed included age, sex, education, household income per annum, body mass index (BMI), occupational status, marital status and presence of chronic disease (presence or absence). These socio-demographic characteristics were collapsed into categories as shown in Table 1. The time since the individual registered with 10,000 Steps was obtained from the registration database.

**Table 1:** Self-rated health, socio-demographic and lifestyle behaviour characteristics of study participants (n = 10,478).

	N = 10,478	%
**Self-Rated Health**		
Poor	1240	11.83
Good	9238	88.17
**Unhealthy Days**		
Frequent Unhealthy Days	2039	19.46
Infrequent Unhealthy Days	8439	80.54
**Gender**		
Female	7390	70.53
Male	3088	29.47
**Age**		
18–34	1842	17.58
35–44	2359	22.51
45–54	3557	33.95
55+	2720	25.96
**Education**		
Secondary School or Less	1703	16.25
TAFE	2339	22.32
University	6436	61.42
**Paid Employment**		
Yes	9303	88.79
No	1175	11.21
**Household Income**		
<$50,000 per year	751	7.17
$50,001–$100,000 per year	3187	30.42
>$100,000 per year	4673	44.60
other/not reported	1867	17.82
**Marital Status**		
Never Married	2928	27.94
Separated/other	1157	11.04
Currently Married	6393	61.01
**Chronic Disease**		
Yes	5196	49.56
No	5282	50.41
**BMI**		
Healthy Weight (≥18.5 - <25)	4051	38.66
Overweight (25 - <30)	3753	35.82
Obese (≥30)	2674	25.52
**Smoking Status**		
Non-smoker	9749	93.04
Current Smoker	729	6.96
**Alcohol Consumption**		
Lower risk	7433	70.94
Higher risk	3045	29.06
**Physical Activity**		
High	5091	48.59
Moderate	3927	37.48
Low	1460	13.93
**Dietary Behaviours**		
Highest Tertile (better diet)	3505	33.44
Middle Tertile	3209	30.63
Lowest Tertile (poorer diet)	3765	35.93
**Sitting Time**		
<8 hrs per day	4228	40.35
8 to <11 hrs per day	4226	40.33
≥11 hrs per day	2024	19.32
**Sleep Duration**		
≥7 to <8 hrs per night	4205	40.13
<7 hrs per night	3979	37.97
≥8 hrs per night	2294	21.89
**Sleep Quality**		
Very Good	1836	17.52
Fairly Good	6188	59.06
Fairly Bad/Very bad	2454	23.42

Note: TAFE is provider of vocational non-bachelor education up to level of advanced diploma.

### Outcome Measures

Health related quality of life was measured using the CDC Healthy Days Instrument, a valid and reliable measure of self-rated health, frequency of unhealthy days in the last 30 days and activity limitation [31]. This instrument has demonstrated acceptable test-retest reliability and validity when compared to the SF-36 Health Survey [32], [33]. Similar to previous research, the current study uses two outcomes from the Healthy Days instrument: self-rated health, dichotomized as *poor* (poor or fair) and *good health* (good, very good or excellent); and unhealthy days, dichotomized as *frequent unhealthy days* (≥14 unhealthy days out of the past 30 days) or *infrequent unhealthy days* (<14 unhealthy days out of the past 30 days) [34]–[36].

### Analysis

Analysis was limited to participants who provided complete data on the socio-demographics, health-related quality of life, and lifestyle behaviors (n = 10,478). Multi-variable Robust Cox regression with a constant time (time = 1) for all participants was used to estimate the prevalence of poor self-rated health and frequent unhealthy days in relation to lifestyle behaviors [37]. Separate analyses were used to examine the prevalence of study outcomes by each lifestyle behavior when adjusting for socio-demographic characteristics and other lifestyle behaviors. To assess the joint impact of lifestyle behaviors, two lifestyle behavior indices were created. In Index 1, each participant scored a single point for each of the following lifestyle behaviors: current smoker, higher risk alcohol, moderate or low physical activity, middle or lower tertile of dietary behaviors, sitting time of ≥8 hours per day, and sleep duration of <7 hours or ≥8 hours per day (possible Index 1 range was 0 to 6). In Index 2, participants scored a single point for each of the behaviors in Index 1 and scored an additional point if they reported fairly good or fairly bad sleep quality (possible Index 2 range was 0 to 7). This approach to creating Lifestyle Behavior Indices allows this study to examine how multiple lifestyle behaviors influence health status and extends similar studies [3] by the inclusion of smoking, alcohol, physical activity, dietary, sitting and sleep duration to be examined in a single index, which few studies have done previously [4], [15]. It further extends knowledge regarding the influence of multiple lifestyle behaviors on health status by examining all these behaviors when sleep quality is also included [19], [38]. Adding sleep quality in a separate index of multiple lifestyle behaviors will help to demonstrate the relative importance of this infrequently examined factor. Due to the low number of participants with a score of zero in each index (Index 1 = 3.14%; Index 2 = 0.73%), participants with a score of 0 or 1 were collapsed into a single category in each index. Separate analyses were then undertaken to examine the prevalence of study outcomes by each index adjusting for socio-demographic characteristics. Full details of the covariates included in each model are detailed in Tables 2 and 3. Analyses were conducted using STATA 12.0 and a p value of 0.05.

**Table 2:** Prevalence of Poor Self-rated Health and Frequent Unhealthy Days Across Different Health Behaviors (n = 10,478).

	Poor Self-rated Health		Frequent Unhealthy Days	
Health Behavior	PR (95% C.I)^1^	p	PR (95% C.I)^1^	p
**Smoking Status**				
Non Smoker	1.00		1.00	
Current Smoker	1.37 (1.17–1.60)	<0.001	1.18 (1.04–1.34)	0.008
**Alcohol Consumption**				
Lower Risk	1.00		1.00	
Higher Risk	0.98 (0.88–1.09)	0.736	0.88 (0.81–0.96)	0.003
**Physical Activity**				
High	1.00		1.00	
Moderate	1.58 (1.40–1.78)	<0.001	1.05 (0.96–1.14)	0.281
Low	2.17 (1.89–2.48)	<0.001	1.34 (1.21–1.48)	<0.001
**Dietary Habits**				
Highest Tertile (better diet)	1.00		1.00	
Middle Tertile	1.11 (0.95–1.29)	0.175	0.99 (0.90–1.10)	0.901
Lowest Tertile (poorer diet)	1.41 (1.23–1.63)	<0.001	1.09 (0.99–1.20)	0.085
**Sitting Time**				
<8 hrs per day	1.00		1.00	
8 to <11 hrs per day	1.14 (1.01–1.28)	0.037	1.15 (1.05–1.25)	0.003
≥11 hrs per day	1.33 (1.17–1.52)	<0.001	1.32 (1.20–1.46)	<0.001
**Sleep Duration**				
7 to <8 hrs per night	1.00		1.00	
<7 hrs per night	1.14 (1.01–1.28)	0.040	1.27 (1.16–1.40)	<0.001
≥8 hrs per night	1.30 (1.12–1.50)	<0.001	1.26 (1.13–1.42)	<0.001
**Sleep Quality**				
Very Good	1.00		1.00	
Fairly Good	1.76 (1.42–2.17)	<0.001	1.75 (1.49–2.06)	<0.001
Fairly/Very Bad	3.03 (2.43–3.79)	<0.001	3.24 (2.73–3.85)	<0.001

Notes: 1. Models were adjusted for length of 10,000 Steps membership, age (18–34; 34–44; 45–54; 55+), gender (male; female), paid employment status (Yes; No), educational level (Secondary School or Less; TAFE; University), annual income (<$50,000 per year; $50,001–$100,000 per year; >$100,000 per year; other/not reported), marital status (Never Married; Separated/other; Currently Married), presence of chronic disease (Yes; No), BMI (Healthy Weight; Overweight; Obese) and all other behaviors listed in the table.

**Table 3:** Association between multiple poor lifestyle behaviors and the prevalence of Poor Self-Rated Health and Frequent Unhealthy Days (n = 10,478).

	Poor Self-Rated Health		Frequent Unhealthy Days	
Lifestyle Behavior Index	PR (95% C.I)^1^	p	PR (95% C.I)^1^	p
**Index 1**				
0–1	1.00		1.00	
2	1.54 (1.19–1.98)	0.001	1.31 (1.12–1.53)	0.001
3	2.07 (1.63–2.63)	<0.001	1.54 (1.33–1.79)	<0.001
4	3.00 (2.36–3.81)	<0.001	1.83 (1.57–2.12)	<0.001
5	3.61 (2.78–4.69)	<0.001	1.98 (1.66–2.36)	<0.001
6	3.89 (2.71–5.60)	<0.001	2.07 (1.49–2.86)	<0.001
**Index 2**				
0–1	1.00		1.00	
2	2.26 (1.25–4.09)	0.007	1.25 (0.94–1.65)	0.129
3	3.29 (1.87–5.81)	<0.001	1.74 (1.34–2.27)	<0.001
4	4.68 (2.66–8.21)	<0.001	2.09 (1.61–2.72)	<0.001
5	6.48 (3.68–11.40)	<0.001	2.57 (1.97–3.33)	<0.001
6	7.91 (4.45–14.06)	<0.001	2.71 (2.05–3.59)	<0.001
7	8.55 (4.59–15.94)	<0.001	2.90 (1.97–4.28)	<0.001

Notes: 1. Models were adjusted for length of 10,000 Steps membership, age (18–34; 34–44; 45–54; 55+), gender (male; female), paid employment status (Yes; No), educational level (Secondary School or Less; TAFE; University), annual income (<$50,000 per year; $50,001–$100,000 per year; >$100,000 per year; other/not reported), marital status (Never Married; Separated/other; Currently Married), presence of chronic disease (Yes; No), BMI (Healthy Weight; Overweight; Obese).

## Results

**Figure 1:**
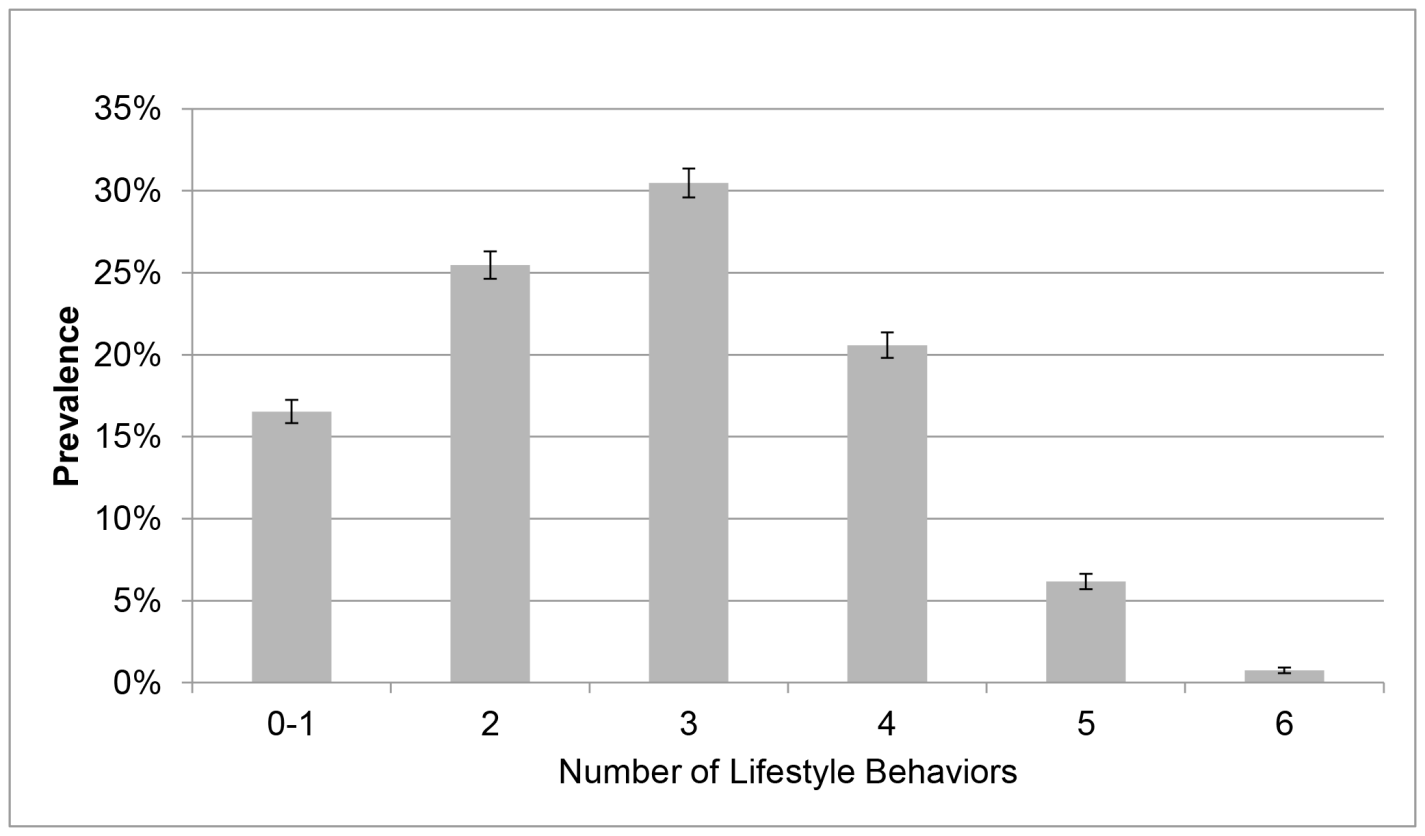
Prevalence of Multiple Poor Lifestyle Behaviors (Index 1). Figure 1 Notes. The proportion of participants reported zero to one, two, three, four, five and six poor health behaviors was 16.54%, 25.47%, 30.47%, 20.59%, 6.17%, and 0.75%, respectively. Unadjusted prevalence and 95% CI.

**Figure 2:**
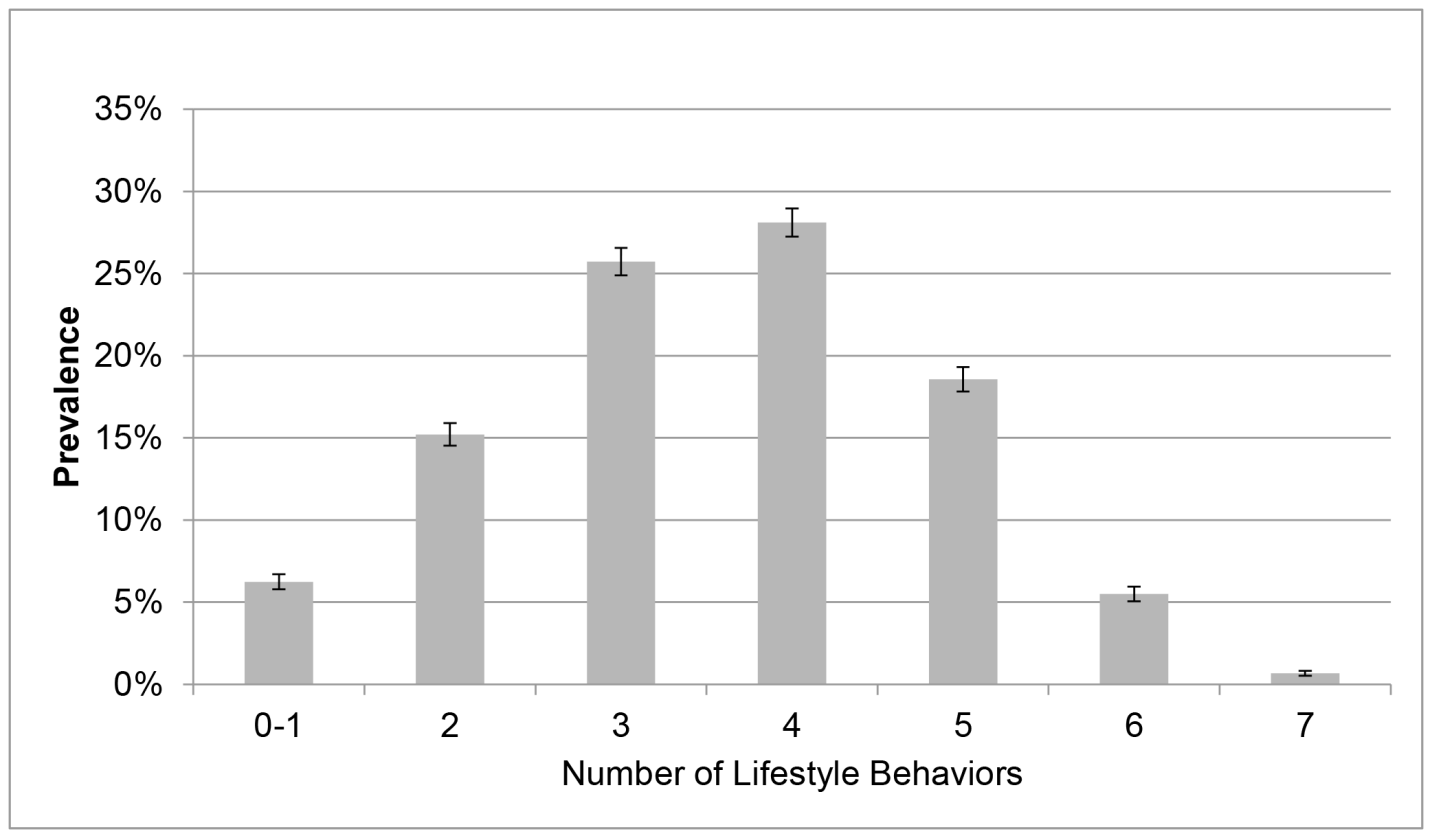
Prevalence of Multiple Poor Lifestyle Behaviors (Index 2). Figure 2 Notes. The proportion of participants reported zero to one, two, three, four, five and six poor health behaviors was 6.24%, 15.20%, 25.72%, 28.10%, 18.57%, 5.50%, and 0.67%, respectively. Unadjusted prevalence and 95% CI.xl.

A total of 14,145 individuals completed the survey providing a response rate of 11.66%. The main reasons for non-response to the email invitation were undeliverable emails (e.g., no longer in use, bounce back, out of office reply) (n = 38,434) and decline to participate (n = 2,106). Due to missing data for variables included in the analysis, the current analysis is delimited to 10,478 individuals. The majority of participants were female (70.53%), aged 45–54 years (33.95%), had a University level of education (61.42%) and were in paid employment (88.79%). Approximately 39% of participants were classified as having a healthy BMI (Table 1). The overall prevalence of poor self-rated health and frequent unhealthy days was 11.83% and 19.46%, respectively. The majority of participants reported being non-smokers, consumed alcohol at lower risk levels and engaged in a high level of physical activity (Table 1). Most participants reported sitting less than 11 hours per day and obtaining fairly good sleep quality. For Index 1, over one-half of participants (57.23%) reported 3–5 poor lifestyle behaviors (Figure 1), which increased to nearly three-quarters of participants (72.39%) for Index 2 (Figure 2).

Table 2 summarizes the associations between individual lifestyle behaviors and health-related quality of life. Current smokers reported a higher prevalence of poor health compared to non-smokers for both outcomes (Table 2). Higher risk alcohol consumption was associated with a lower prevalence of frequent unhealthy days compared to lower risk alcohol consumption. Lower levels of physical activity were associated with a higher prevalence poor self-rated health; however, the association between physical activity and frequent unhealthy days was only significant for low compared to high levels of physical activity. Participants reporting the poorest quality diet had a higher prevalence of poor self-rated health; however, there was no association between the prevalence of frequent unhealthy days and dietary behaviors. Compared to sitting for 8 hours or less per day, sitting for 8 to 11 hours per day and sitting for more than 11 hours per day was associated with poor self-rated health and frequent unhealthy days. Compared to sleeping 7 to 8 hours per night, sleeping <7 hours per night and sleeping ≥8 hours per night was associated with a higher prevalence of poor health for both outcomes. Compared to participants reporting very good sleep quality, reporting fairly good and fairly bad sleep quality was associated with a higher prevalence of both outcomes.

Table 3 shows that the prevalence of poor self-rated health and frequent unhealthy days increases with the number of poor lifestyle behaviors reported. For Index 1, compared to reporting 0–1 poor lifestyle behaviors, participants reporting two poor lifestyle behaviors had a higher prevalence of poor self rated health (PR = 1.54, 95% CI: 1.19–1.98) and frequent unhealthy days (PR = 1.31, 95% CI: 1.12–1.53). Participants reporting the maximum number of poor lifestyle behaviors in Index 1 (six behaviors) had a higher prevalence of poor self-rated health (PR = 3.89, 95% CI: 2.71–5.60) and frequent unhealthy days (PR = 2.07, 95% CI: 1.49–2.86). For Index 2, participants reporting two poor lifestyle behaviors had a higher prevalence of poor self-rated health (PR = 2.26, 95% CI: 1.25–4.09) but not frequent unhealthy days (PR = 1.25, 95% CI: 0.94–1.65). The prevalence of poor self-rated health (PR = 8.55, 95% CI: 4.59–15.94) and frequent unhealthy days (PR = 2.90, 95% CI: 1.97–4.28) was higher in participants reporting the maximum number of poor lifestyle behaviors (seven behaviors) in Index 2 compared to participants reporting 0–1 poor lifestyle behaviors. The prevalence of poor self-rated health is notably higher when sleep quality is considered as a lifestyle behavior.

## Discussion

This study demonstrated that smoking, lower levels of physical activity, poor dietary behaviors, higher sitting time, and poor sleep behaviors were independently associated with a higher prevalence of poor self-rated health and frequent unhealthy days. Furthermore, a greater number of poor lifestyle behaviors were associated with a higher prevalence of poorer health. The outcomes of this study extend similar examinations of multiple lifestyle behaviors [3], [4], [13]–[15] by demonstrating that sleep quality exacerbated the association between unhealthy behaviors and health outcomes. This finding is a novel and important aspect of this study given most participants reported less than optimal sleep quality and that 75% of participants reported engaging in more than 3 poor lifestyle behaviors. It also reinforces the notion that reducing the number of people engaging in multiple unhealthy behaviors is an important public health objective [3], [4], [15].

An individual’s risk of poor health increases when they engage in multiple unhealthy lifestyle behaviors [1], [4], [39]. The results of this study, demonstrating that individual poor lifestyle behaviors and each additional poor lifestyle behavior is associated with poorer health, provides strong rationale to intervene on multiple lifestyle behaviors at the population level [5], [40], [41]. There is good evidence regarding the effectiveness of interventions targeting both improvements in physical activity and dietary habits to reduce CVD risk [42] and a large literature base discussing the theoretical, conceptual and methodological issues associated with conducting multiple behavior change interventions [43]–[45]. Although debate exists whether sequential or simultaneous interventions are more effective in changing behaviors [44], interventions have been effective in changing two [46], [47], three [48], and four or more behaviors simultaneously [49], suggesting that it may be possible to intervene on all the lifestyle behaviors examined in the current study. However, as others have noted [39], [50], the challenge lies in disseminating such an intervention to large number of individuals at acceptable cost.

The findings regarding sleep duration and poorer health are consistent with other studies demonstrating that sleeping less than 7 hours per day or sleeping more than 8 hours per day is associated with poorer self-rated health, future risk of type 2 diabetes and increased all-cause mortality [4], [51], [52]. The current study extends these observations by demonstrating that sleep quality is also associated with the prevalence of poor self-rated health when adjusting for a wide range of lifestyle behaviors previously linked to poor self-rated health [1], [16], [53], [54]. It is noteworthy that, compared to other lifestyle behaviors, poor sleep quality was associated with the highest prevalence of poor self-rated health in the current study. Although in some ways a modifiable voluntary behavior, sleep quality is also influenced by aging, chronic health conditions, and obesity [55], [56]. Although the current study statistically adjusted for age, chronic disease status, and BMI, it may be that covariate control was inadequate to fully account for the complex influence of these factors on the relationship between sleep quality and self-rated health [52]. Prospective studies that enable stratified analysis by these factors are needed to disentangle this relationship [52]. Alternatively, it may be that the negative psychophysical consequences of poor quality sleep are sufficient to lower individuals’ perceived health status even after statistically adjusting for other lifestyle behaviors. Further, evidence indicates that physical activity may improve sleep quality [55], [56] Thus, it may be that interventions targeting multiple lifestyle behaviors may enhance the individual behaviors and also create synergistic improvements in targeted behaviors [44].

Consistent with previous studies [54], [57], the current study demonstrated that longer duration of sitting time is associated with poorer self-rated health and extends those studies by demonstrating this relationship when accounting for a number of other lifestyle behaviors including sleep. Sleep behavior is seldom accounted for when examining relationships between sitting and health status despite its impact on health status [4], [5], [15], [40]. As such, this association is a useful contribution to this literature. The prevalence of poor self-rated health and frequent unhealthy days in the current study is comparable to other studies of the general population in other countries, including Australia, suggesting that this population was not biased by higher levels of self-rated health [36], [39]. The association between higher alcohol intake and the lower prevalence of frequent unhealthy days is both in agreement with and in contrast to other studies and may be due to the volumes of alcohol consumed by participants, their pattern of consumption or other unmeasured factors [14], [58]. As this study population had a high proportion of middle-aged females and participants from a higher socio-economic status (based on education and income) it would be useful to confirm if the presence of the associations observed in this study are present in more representative populations. Further limitations to the current study are a reliance on self-report measures of lifestyle behaviors and its cross-sectional nature. The response rate to the current survey was modest compared to other online surveys [59], and was likely impacted by the number of undeliverable emails which is a function of the long period of time that people have been able to register on the 10,000 Steps website. These limitations are offset by the strengths of the study, including the sample size, examination of health indicators that are strongly linked to chronic disease risk and mortality [31], [35], assessment of behaviors and outcomes using measures with good psychometric properties, and the range of lifestyle behaviors examined. Currently there are few studies that have examined sleep duration and quality in conjunction with other lifestyle behaviors such as physical activity, diet and sitting that are more commonly examined in the public health context. This is a significant strength of the current study given the growing recognition of sleep behavior as an important lifestyle behavior to assist in reducing the prevalence of chronic diseases and other public health issues [5], [60]. Furthermore, examining the role of sleep quality is an important extension of previous studies that have only examined sleep duration as a risk factor of poor health.

In conclusion, this study demonstrated that, excluding alcohol intake, each of the poor lifestyle behaviors examined was associated with poor health status and that the prevalence of poor health increased as the number of poor lifestyle behaviors increased. Furthermore, associations when examining the combined influence on unhealthy lifestyle behaviors were exacerbated when sleep quality was included in the index, indicating the importance of sleep quality in maintaining good self-rated health.
